# Lead (Pb) and the NMDA Receptor Hypofunction Hypothesis: A Comparative Review of Non-Essential Metal Exposures in Schizophrenia

**DOI:** 10.1007/s40572-026-00551-9

**Published:** 2026-07-13

**Authors:** Jennifer S Smith, Kendall P Dean, Justin A Colacino, Dana C Dolinoy, Alexis J Handal

**Affiliations:** 1 Department of Environmental Health Sciences, University of Michigan School of Public Health, Ann Arbor, MI 48105 USA; 2 Cellular and Molecular Biology Program, University of Michigan Medical School, Ann Arbor, MI 48109-5632 USA; 3 Department of Epidemiology, University of Michigan School of Public Health, Ann Arbor, MI 48105 USA

**Keywords:** Schizophrenia, NMDA receptor, Metals, Lead (Pb), Environmental exposure, Glutamate

## Abstract

**Purpose of Review:**

Schizophrenia (SCZ) is a debilitating neuropsychiatric disorder characterized by hallucinations, delusions, paranoia, and anhedonia [1, 2]. Strong biological plausibility supports a disease-specific relationship between metal and metalloid exposures and SCZ, particularly through disruption of metal ion coordination within with the N-methyl-D-aspartate receptor (NMDAr), a key regulator of neurodevelopment and synaptic plasticity [3–5]. This review synthesizes existing epidemiological evidence linking metal exposure to SCZ and integrates these findings with longstanding hypotheses implicating NMDAr hypofunction as a core neurobiological mechanism underlying the disorder.

**Recent Findings:**

Fifteen studies met inclusion criteria, including thirteen identified through systematic database searches and two identified through external sources. Overall, findings demonstrated positive associations between metal exposures and schizophrenia-related outcomes. Lead (Pb) emerged as the most consistently associated metal across studies (associated with SCZ in 8 of 10 studies evaluating Pb). Additional metals and metalloids associated with SCZ included Cd (associated with SCZ in 4 of 9 studies evaluating Cd), Cr (associated with SCZ in 3 out of 5 studies evaluating Cr), U (associated with SCZ in 2 of 2 studies evaluating U), and As (associated with SCZ in 2 of 6 studies evaluating As). The following metals were each also associated with SCZ in 1 of 1 study evaluating them: W, Sb, Ba, and Cs. Dysregulation of essential metals including Se, Fe, Cu, Ca, Mn, and Zn were also associated with SCZ, suggesting that both toxic metal burden and disruption of essential metal homeostasis may contribute to disease risk.

**Summary:**

Current epidemiological evidence supports an association between metal biomarker levels and SCZ, with Pb most consistently implicated. These findings may be consistent with the NMDAr hypofunction hypothesis. However, inconsistent exposure assessment, variable diagnostic criteria, and limited consideration of developmental timing constrain causal inference. Future research incorporating standardized exposure metrics, improved temporal resolution, and gene–environment frameworks is needed to clarify causal relationships and identify vulnerable populations.

## Introduction

Metals and metalloids (hereafter referred to as “metals”) are a persistent public health concern due to their ubiquity and adverse effects on human and ecological health [1–3]. Exposure to metals can take place through a combination of anthropogenic and natural means. Anthropogenic sources for metal exposures often include mining, waste disposal, fertilization and pesticides, industrial processes, agriculture and forestry, and fossil fuel combustion [4]. For example, mining for metals like gold often utilizes mercury in its purification process, while mining for uranium is its own industry and often a devastatingly toxic polluter of underserved populations, namely the Navajo Nation and other tribal communities [5]. Although leaded gasoline was phased out in the U.S. by the late 1980s, historical emissions remain a significant source of anthropogenic lead exposure, as legacy lead persists in urban soils due to decades of vehicular exhaust and past use of lead paint depositing lead particles into the environment [6, 7]. Today, approximately half of the United States population has been exposed to a combination of two or more metals, the most prevalent being combinations of Pb/Cd, Pb/Cd/Hg/As, and Pb/Cd/Hg [8].

Exposure to neurotoxic metals such as Pb, As, Cd, Hg, and others have been implicated in adverse cognitive and psychiatric outcomes across the lifespan [3, 9, 10]. However, precisely how and which metal exposures contribute to specific psychiatric disease phenotypes, such as schizophrenia, is poorly understood. Schizophrenia (SCZ) is a severe psychiatric disorder affecting ~ 1% of the global population, often leading to long-term disability, reduced life expectancy, high unemployment, and substantial societal costs [11–13]. The disease is characterized by positive symptoms, including auditory and visual hallucinations, and negative symptoms, such as apathy and anhedonia [14]. Disease pathoetiology for SCZ is poorly understood despite multiple proposed candidate hypotheses, including genetic, epigenetic, and environmental origins [15].

Genetic polymorphisms in genes including *GRIN2A*, which encodes the NR2A subunit of the N-methyl-D-aspartate receptor (NMDAr) and *GRIN2B*, which encodes the NR2B subunit of the NMDAr have been associated with increased risk of developing SCZ during adolescence or adulthood [16–18]. NMDArs are ionotropic, heterotetrametric, glutamate receptors composed of two obligatory NR1 subunits alongside two regulatory subunits from either the NR2 (A–D) or NR3 (A–B) families, encoded by the *GRIN1*, *GRIN2A–D*, and *GRIN3A–B* genes, respectively. In the hippocampus and prefrontal cortex, NR2A and NR2B subunits are most abundantly expressed. When activated by glutamate and co-agonists like glycine or D-serine, NMDArs mediate Ca influx in the post-synaptic neuron after dislodging Zn from an allosteric binding pocket and Mg from the calcium channel, forming the basis of excitatory neurotransmission, long term potentiation, and plasticity [19, 20]. Impaired excitatory neurotransmission and NMDAr hypofunction has been observed in multiple psychiatric disorders, especially SCZ [21]. One candidate reason for NMDAr hypofunction in SCZ in the context of environmental exposures could be due to the reliance of the NMDAr on coordinated metal ions to function. NMDArs directly rely on Zn, Ca, and Mg, and indirectly rely on homeostasis of other essential metal ions like Cu and Fe for overall neuron health and development [22, 23]. Non-essential metals from environmental exposures such as Pb, Cd, Hg, Cr, As, and others, have the ability to mimic and dysregulate Ca and Zn. The tendency for Pb specifically to bind to proteins meant to bind Ca and Zn has been well documented presumably due to its similar atomic size to Ca (~ 1.19 Å for Pb versus 1.00 Å for Ca), and divalent cation properties like Ca and Mg [24].

While multiple environmental triggers have been presented and explored in recent decades, including maternal influenza infection during pregnancy, gestational micronutrient deficiencies, heavy cannabis use during adolescence, exposure to *Toxoplasma Gondii*, no single environmental exposure has been definitively shown to cause SCZ [25–28]. Furthermore, many proposed explanations for the disorder are limited to either molecular-level mechanisms or population-level epidemiological patterns. The convergence of existing research connecting NMDAr dysfunction to SCZ and the biological potential for environmental factors to negatively influence the NMDAr via metal dysregulation warrants investigation into the association between non-essential metals and SCZ specifically.

This review synthesizes existing epidemiological evidence linking metal exposure to SCZ and integrates these findings with the longstanding hypotheses implicating NMDAr hypofunction as a core neurobiological mechanism underlying the disorder. To determine whether specific metals are consistently associated with SCZ, a systematic search was conducted using PubMed and Google Scholar with defined MeSH terms. This yielded 15 studies that met the inclusion and exclusion criteria. Across these studies, epidemiological evidence supported associations between metal exposure and SCZ, with lead (Pb) emerging as the most consistently implicated metal in patients with SCZ. Compared to other metals, Pb’s common environmental prevalence, its unique distribution and storage kinetics, and its capacity to inhibit NMDA receptor (NMDAr) signaling may render it uniquely positioned to influence SCZ risk. Therefore, the second section of this review explores potential molecular mechanisms linking Pb exposure to SCZ, with a particular focus on the vulnerabilities of the N-methyl-D-aspartate receptors (NMDArs) to Pb interference.

Taken together, this review observes emerging patterns linking epidemiological associations between Pb exposure and SCZ with plausible molecular mechanisms underlying disease pathoetiology. Further research ought to be conducted to understand how genetic predisposition to SCZ might interact with environmental exposures to contribute to disease exacerbation and onset.

## Methods

Key concepts ‘SCZ’, ‘Heavy Metals’, and ‘Environmental Exposure’ were used to generate MeSH terms in the PubMed database. The final MeSH term search included, ( “Heavy Metal Poisoning, Nervous System“[Mesh] OR “metals, heavy“[MeSH] OR “metalloids“[Mesh] OR Lead[MeSH] OR mercury[Title/abstract] OR cadmium[Title/abstract] OR chromium[Title/abstract] OR arsenic[Title/abstract] OR aluminum OR nickel[Title/abstract] OR uranium[Title/abstract] OR titanium[Title/abstract] OR thallium[Title/abstract] OR tungsten[Title/abstract]) AND ( “schizophrenia” [Mesh] ) NOT ( “Review“[Publication Type] OR “Systematic Review“[Publication Type] OR “Meta-Analysis“[Publication Type] OR “Editorial“[Publication Type] OR “Comment“[Publication Type] OR “Letter“[Publication Type] OR “Case Reports“[Publication Type] OR “Med Hypotheses“[Journal]). Search filters were applied to include only human data, and only epidemiological data was included in the final dataset, excluding human-derived in vitro experiments. The initial segment of the search query (all terms preceding the AND operator) was designed to identify studies investigating at least one biologically non-essential metal. The metals included were selected based on prior evidence demonstrating their classification as environmental pollutants derived predominantly from anthropogenic activities. The second portion of search terms was used to generate results strictly examining SCZ as the disease outcome of interest. The third portion of search terms was included to ensure that the literature yielded from the search was only primary data, and any reviews, editorials, opinion pieces, or any other type of literature not involving the collection and processing of original data were excluded. Additionally, case reports were excluded in this portion of the search due to their low sample size (typically *n* = 1) and often exceptional results not widely generalizable to a population.

## Results

**Fig. 1:**
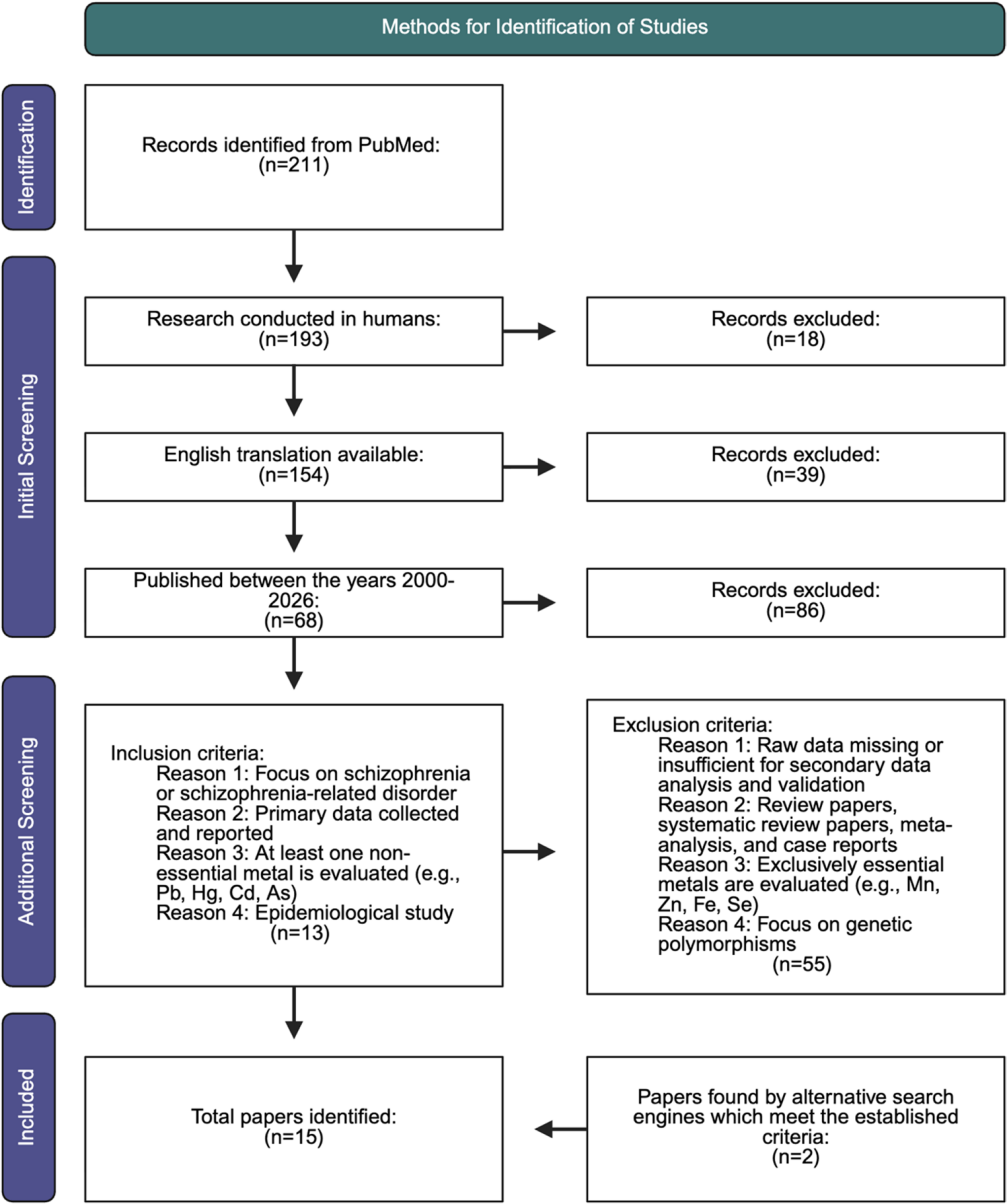
Flow diagram of study identification and selection. Studies were identified through PubMed and Google Scholar and screened based on language, publication year, human subjects, and relevance to metal exposure and schizophrenia-spectrum disorders. 13 studies met inclusion criteria, including two identified through alternative search engines

The full search term yielded 211 results (Fig. 1). After filtering results to only include human subjects, there were 193 articles. After filtering results to only include articles with English translations available, there were 154 articles. After restricting results to only include articles written between the years 2000–2026, there were 68 articles. Upon application of inclusion and exclusion criteria, there were 13 articles [14, 29–41] and two additional articles were found through external search engines that met inclusion criteria [9, 42]. One additional study by Nawaz et al., [43] was identified through external search engines but was ultimately excluded due to its use of siblings of patients with SCZ as the control group. Given the elevated lifetime risk of SCZ among first-degree relatives compared to the general population, the use of sibling controls introduced potential genetic and environmental confounding, limiting the appropriateness of the comparison [44].

Inclusion criteria included the following: Human studies, English translation available, peer-reviewed articles from the years 2000–2026 (no preprints), a focus on SCZ as the primary disease outcome, primary data collected and reported upon, thereby excluding reviews, editorials, and journals, and at least one non-essential metal was evaluated (Pb, Hg, Cd, Cr, As, Al, Ni, U, Ti, Tl, W). Exclusion criteria were as follows: studies that analyzed only biologically essential metals (*n* = 24); studies in which genetic polymorphisms were the primary focus (*n* = 7); studies centered on clinical assay validation (*n* = 7); studies in which metal exposure was not a primary focus (*n* = 7); studies primarily evaluating antipsychotic treatment effects (*n* = 3); and studies examining non-SCZ disease outcomes (*n* = 3).

Three additional studies were excluded for study-specific reasons. These three studies were Stanley et al., [45], Sallmén et al., [46] and Todorić et al., [47]. Stanley et al., [45] was excluded due to insufficient data and supplementary tables. Sallmén et al., [46] was excluded due to its nature being a paternal exposure and not a direct exposure to the patient with SCZ. This retrospective cohort study, conducted in Finland with a large sample size (*N* = 11,863), examined the relationship between paternal lead exposure and increased risk of SCZ spectrum disorders in offspring. While methodologically robust, it differs from the other studies reviewed here, which examined metal exposures directly to the study participants. As such, its findings are more applicable to questions of transgenerational toxico-epigenetic inheritance via the paternal germline—an important area of inquiry beyond the scope of this review [48]. Todorić et al., [47] was excluded due to the absence of a healthy control group for comparison of body metal burden. This study instead evaluated associations between scalp hair metal concentrations and symptom severity within a SCZ cohort. The severity of distinct symptoms within SCZ according to metal burden is an important area of research outside of the scope of this review. Additional papers (*n* = 3) were found on Google Scholar that met inclusion and exclusion criteria.

### Study Designs and Characteristics

Of the 15 articles included in the final literature review, six took place in China [31, 33–36, 38] three took place in the United States [29, 30, 32], two took place in Bangladesh [40, 42], and one took place in Nigeria [9], Iran [37], India [39], and Iraq [41], respectively (Table 1).

**Table 1:** Summary of studies included in final analysis. Studies by Ghaderi et al., [37], Liu et al., [31], and Karim et al., [42] did not identify associations between non-essential metal exposures and SCZ. All other studies listed identified an association between non-essential metal exposures and SCZ, eight of which identified Pb as most associated with SCZ. Within the cross-sectional case control studies, the three values represent for Ghaderi et al., [37], 56 smokers w/o SCZ, 58 smokers w/ SCZ, 57 non-smokers without SCZ, Arinola et al., [9] 20 medicated SCZ patients, 15 newly diagnosed, unmedicated SCZ patients, and 20 controls. Instrument and analytical method abbreviations include graphite atomic absorption spectrophotometry (GFAAS), inductively coupled plasma mass spectrometry (ICP-MS), laser ablation inductively coupled plasma mass spectrometry (LA-ICP-MS), atomic absorption spectrophotometer (AAS), flame atomic absorption spectroscopy (FAAS), LC-40 fluorescence detection (LC-40 FD)

Country	Study	Sample size (*n*)	Study design	Cases (SCZ diagnosis +): Control	Covariates adjusted	Biological specimen	Instrument/Analytical method	Metals analyzed	Metals significantly associated with SCZ	OR, CI, or pvalue
India	Ray et al., [39]	80	Cross-sectional case control	40:40	Stroop test performance, TNF-α and HM levels	Adult whole blood and serum	GFAAS	Pb, As, Al, Cd	↑ Pb, Cd, ↓ As	Pb: *p* = 0.015 Cd: *p* = < 0.001 As: *p* = 0.01
China	Li et al., [36]	440	Cross-sectional case control	221:219	Age, sex	Adult blood serum	ICP-MS	Ba, W, U	↑ W, U	W Q4: OR 1.87 (95% CI 1.08–3.21) U Q2: OR 2.06 (1.19–3.56), U Q3: OR 1.99 (1.15–3.44), U Q4: OR 1.74 (1.00–3.00)
China	Shen et al., [38]	440	Cross-sectional case control	221:219	Age, sex	Adult blood serum	ICP-MS	Pb, As, Cd, Cr	↑ Pb	OR 3.62 (95% CI: 1.80–7.28) for Q4 compared to Q1
Iran	Ghaderi et al., [37]	171	Cross-sectional case control	56:58:57	None	Adult blood serum, urine for Tl	GFAAS	Tl, As, Cd, Pb	None (no direct association demonstrated)	None reported (no ORs)
Iraq	Almulla et al., [41]	174	Cross-sectional case control	120:54	Age, sex, BMI, tobacco use disorder (TUD), employment, income, urban/rural residence, marital status	Adult blood serum	GFAAS	Cs, Rb, Re	↓ Cs	*p* = 0.003
China	Ma et al., [34]	190	Cross-sectional case control	95:95	Marital status	Adult blood serum	ICP-MS	Cr, Cd, Pb, As	↑ Pb, ↑ Cr in later meta-analysis	Pb: OR 3.15 (1.24–7.99), Cr: SMD = 0.41 (95% CI 0.10–0.73)
China	Ma et al., [35]	215	Cross-sectional case control	109:106	Lanthanum (La), age, sex, BMI, marital status, migration experience, family income, smoking, drinking, and adverse psychological stimulation	Adult blood serum	ICP-MS	Hg, Pb, Cr, Ag, Sb, U	↑ Sb, U	Sb: OR 8.03 (3.23–19.93) U: OR 2.16 (1.07–4.39)
China	Li et al., [33]	827	Cross-sectional case control	158:669	None	Adult blood serum	ICP-MS, FAAS	Zn, K, Ca, Mg, Cu, Fe, B, Mn, Se, Cr, Cd, Pb and As	↑ B, Cr, As, K, Mg ↓ Mn, Se, Cd, Pb, Ca, Cu and Fe ↑ Pb in < 29 y/o	As: OR 2.624 (95% CI 1.340–5.138), *p* = 0.005 K: OR 1.035 (1.007–1.064), *p* = 0.015 Cr: OR 0.417 (0.182–0.955), *p* = 0.039 Mn: OR 0.068 (0.007–0.655), *p* = 0.020 Se: OR 0.955 (0.918–0.994), *p* = 0.024 Ca: OR 0.971 (0.943–0.999), *p* = 0.046 Cu: OR 0.009 (0.000–0.263), *p* = 0.006 Pb (< 29 y/o): *p* = 0.037
United States, Netherlands	Modabbernia et al., [32]	14	Retrospective case-control	9:5	None	Childhood tooth enamel	LA-ICP-MS	Pb, Mn, Cd, Cu, Mg, Zn	↑ Early-life Pb ↓ Postnatal Mn, Cu	None reported (no ORs), early-life Pb significant after Bonferroni correction
China	Liu et al., [31]	228	Cross-sectional case control	114:114	Age, sex (matched); multivariable model adjusted for significant trace elements only (no BMI, smoking, diet, medications)	Adult blood serum	ICP-MS	Ni, Mo, As, Al, Cr, Mn, Se, Cu, Fe, Zn	↑ Mn, ↓ Se, Cu	Mn: OR 19.269 (95%CI: 1.436–258.626) Se: OR 16.837, 95%CI: 2.130–133.113) Cu: OR 20.957 (95%CI: 1.381–318.141)
Nigeria	Arinola et al., [9]	55	Cross-sectional case control	20:15:20	None	Adult blood serum	AAS	Se, Zn, Fe, Mg, Mn, Cu, Pb, Cd, Cr	Unmedicated, newly diagnosed: ↑ Pb, Medicated: ↑ Zn Both: ↑ Cd, Cr, ↓ Fe, Se	↓ Fe: *p* < 0.001 (drug-naïve vs. control); *p* < 0.001 (medicated vs. control) ↓ Se: *p* < 0.001 (drug-naïve vs. control); *p* < 0.001 (medicated vs. control) ↑ Pb: *p* < 0.001 (drug-naïve vs. control) ↑ Cd: *p* < 0.001 (drug-naïve vs. control) ↑ Cr: *p* < 0.001 (drug-naïve vs. control) ↑ Zn: *p* < 0.001 (medicated vs. control) ↓ Mn: *p* < 0.001 (medicated vs. control)
Bangladesh	Rahman et al., [40]	60	Cross-sectional case control	30:30	None	Scalp hair	FAAS	Mn, Zn, Ca, Cu, Cd	↑ Cu, Cd, ↓ Zn, Ca	Zn: *p* = 0.024 Ca: *p* < 0.001 Cu: *p* = 0.021 Cd: *p* < 0.001
United States	Opler et al., [30]	200	Nested case-control within two prospective birth cohorts	71:129	Maternal age, maternal education	Second-trimester maternal serum (δ-ALA as a biomarker of prenatal lead exposure)	LC-40 FD	Pb (indirectly via δ-ALA)	↑ prenatal δ-ALA— corresponding to ↑ Pb	OR 1.92 (95% CI 1.05–3.52)
Bangladesh	Karim et al., [42]	60	Cross-sectional case control	30:30	None	Adult blood serum	FAAS	Fe, Cd, Pb, Ca	↑ Fe	*p* < 0.001
United States	Opler et al., [29]	119	Nested case-control within prospective birth cohort	44:75	Maternal age	Second-trimester maternal serum (δ-ALA as a biomarker of prenatal lead exposure)	LC-40 FD	Pb (indirectly via δ-ALA)	↑ prenatal δ-ALA—corresponding to ↑ Pb	Unadjusted: OR 1.83 (95% CI 0.87–3.87**)** Adjusted: OR 2.43 (95% CI 0.99–5.96**)**

Several studies utilized established analytical methods for trace metal quantification, including inductively coupled plasma mass spectrometry (ICP-MS), graphite furnace atomic absorption spectrometry (GFAAS), and flame atomic absorption spectrometry (FAAS). ICP-MS is generally considered the most sensitive and rigorous technique for detecting trace metals in biological matrices such as blood or serum, followed by GFAAS [49, 50]. In contrast, FAAS is less sensitive to human blood serum samples and is typically used for metals present at higher concentrations, particularly in environmental samples [51]. Of note, ICP-MS has been shown to be more sensitive to Pb in blood serum than Cd [50].

The studies by Li et al., [36], and Shen et al., [38] utilize the same case-control cohort (*N* = 440). These two studies are distinct due to their investigation of different metals and application of separate analytical frameworks. While Shen et al., [38] examined Pb, Cd, As, and Cr, focusing on mechanistic analyses, Li et al., [36] focused on barium (Ba), tungsten (W), and uranium (U), emphasizing mixture effects and dose-response relationships.

Additionally, the study by Opler et al., [30] is a follow-up study from Opler [29]. These studies used a nested case-control design within prospective birth cohorts to investigate the relationship between prenatal Pb exposure and later development of SCZ Both leveraged archived maternal serum samples collected during the 2nd or 3rd trimester were analyzed from U.S. birth cohorts initiated between 1959 and 1966 and quantified delta-aminolevulinic acid levels (δ-ALA) as a proxy for Pb exposure. Opler et al., [29] focused on the California-based Prenatal Determinants of SCZ (PDS) birth cohort identifying SCZ cases through adult psychiatric interviews and archived prenatal maternal serum samples. Opler et al., [30] extended this work by adding an independent cohort from the New England sites of the New England cohort of the National Collaborative Perinatal Project (NE-NCPP), applying the same exposure and diagnostic methods. A pooled multilevel logistic regression accounted for cohort differences. While methodologically aligned, the studies were treated separately due to the inclusion of new participants from a distinct population in 2008.

### Main Findings

Of the 15 studies included in this review, 12 reported a significant association between non-essential metals in biological samples and SCZ [9, 29, 30, 32–36, 38–41]. Among the included studies, ten assessed Pb in biological samples. Of these, eight identified a positive association between Pb exposure and SCZ, representing the highest number of positive findings among the non-essential metals evaluated in this review [9, 30–32, 32–34, 38, 39]. Of note, one study identified a significantly lower levels of Pb in patients with SCZ compared to controls, but when stratifying by age, found a significantly higher association of Pb in patients with SCZ < 29 y/o [33]. Additional metals and metalloids associated with SCZ included Cd (associated with SCZ in 4 of 9 studies evaluating Cd), Cr (associated with SCZ in 3 out of 5 studies evaluating Cr), U (associated with SCZ in 2 of 2 studies evaluating U), and As (associated with SCZ in 2 of 6 studies evaluating As). W, Sb, Ba, and Cs were also found to be significantly associated with SCZ in one study each.

Several associations between essential metal imbalances and SCZ were additionally noted **(**Table 1**).** Three studies report decreased Se [9, 31, 33]. Two studies report increased Fe in blood serum [9, 33] while one study reported dramatically decreased Fe in blood serum in SCZ patients [42]. Two studies reported decreased Cu [31, 33] while one reported increased Cu in SCZ patients [40]. Two studies reported decreased Ca in SCZ patients [33, 40]. One study saw an increase in Mn and another a decrease in Mn [33]. Only study saw a change in Zn levels [40].

The studies that did not observe an association between non-essential metal exposures and SCZ included those from Ghaderi et al., [37], Liu et al., [31], and Karim et al., [42]. Notably, Liu et al., [31] examined 10 trace elements, three of which were non-essential (As, Al, and Cr), and reported no association between these metals and SCZ. However, the authors observed elevated manganese (Mn) levels in individuals with SCZ. Increased Mn concentrations have been previously associated with psychotic symptoms and SCZ in existing literature [52, 53]. Although Mn is a biologically essential element, excessive exposure—particularly from anthropogenic sources—has been well documented to produce neurotoxic effects [53–55]. Future research that systematically explores the role of essential metal toxicity and SCZ is warranted to better understand this relationship.

## Discussion

### Confounding and Bias

Metals have distinct properties from one another that affect their absorption, distribution, metabolism, excretion, and storage. These properties change vastly depending on age and nutrition [56, 57]. Additionally, the length of time between diagnosis and potential for exposure can be highly relevant when determining associations between metals, especially Pb, given its sequestration into bone tissue after long periods of time have lapsed from exposure [58, 59].

Absorption of lead (Pb) via ingestion may be enhanced by various dietary factors, including a low Ca, Fe, and Zn diet [56]. Infants and young children retain approximately 50% of ingested Pb, reflecting substantially higher gastrointestinal absorption compared to adults [59–62]. Upon entrance into the bloodstream, the vast majority of Pb is bound to erythrocytes and a small fraction remains in the plasma prior to target organ accumulation in the brain, kidneys, liver, and bone [63, 64]. Bone houses Pb for long term storage for up to 30 years due to its effective mimicry of Ca [65, 66]. Specifically, within ~ 1 month of exposure, 90% of Pb is sequestered in bone tissue, 2–8% in various soft tissues, and 2–5% remains in circulating blood [60]. Remobilization of bone Pb can take place during pregnancy, lactation, osteoporosis, resorption (beginning ~ 60–70 years of age) and severe infection, leading to potential for endogenous re-exposure [26, 57–59, 67, 68]. Consequently, Pb measurements in whole blood and serum provide different estimates of Pb exposures: whole blood Pb is frequently regarded as a biomarker of recent exogenous or endogenous exposure due to erythrocyte binding, whereas serum Pb represents the smaller, exchangeable fraction and may better capture Pb mobilized from bone stores [69–71]. Pb is excreted in adults primarily through the urine, followed by some excretion through feces. A major facilitator of Pb excretion is through non-specific binding of cysteine rich proteins, such as glutathione, due to Pb’s high affinity for thiols [72, 73]. A minor fraction of Pb (~ 8%) is eliminated through keratinized tissues, including hair and nails. Collectively, these distinct properties of Pb as an environmental toxicant make the timing, age, and type of biological sampling important to consider in data interpretation of epidemiological evidence. Studies that fail to account for the absorption, distribution, metabolism, excretion, and storage properties of the metals studied are vulnerable to confounding. Contrary to the majority of studies which utilized blood serum in their analyses, Ray et al., [39] utilized whole blood samples to perform their metal concentration quantifications for Pb and As, potentially reflecting a more recent or ongoing Pb exposure [69, 70]. A major limitation of this study is the lack of information regarding the age, sex, and length of time between sampling and diagnosis.

Alternatively, Li et al., [33] did record the average time between SCZ diagnosis and bio-sampling (12.5 years), and Arinola et al., [9] stratified their findings in their collected biosamples by newly diagnosed and previously diagnosed and medicated patients. In the case of Arinola et al., [9] this stratification provided meaningful differences in the findings; significantly elevated Pb was only found in the newly diagnosed, unmedicated SCZ group, relative to control and the medicated SCZ group. In Li et al., [33], elevated blood serum Pb levels were observed in the younger, more recently diagnosed subgroup (< 29 years), but not in older age groups. One possible explanation is progressive sequestration of Pb in bone over time following exposure, which may reduce circulating Pb concentrations in chronic cases. Given that neuroinflammation is strongly implicated in SCZ and that Pb exposure is known to induce neuroinflammatory responses, future research should explore whether acute elevations in Pb may contribute to triggering disease onset in genetically susceptible individuals, with circulating levels potentially declining over time due to redistribution and accumulation in bone and brain tissue [74–76].

Another potential confounding factor in metal quantification from bio sampling is smoking. Smoking status, and more specifically, variability in cigarette metal content, may substantially confound observed associations between metal levels and SCZ. Ghaderi et al., [37] investigated whether blood serum levels of Pb, As, and Cd and urine levels of Tl differed between cigarette smokers diagnosed with SCZ, cigarette smokers without diagnosed SCZ, and non-smoking healthy controls, and whether metal levels were associated with smoking duration. Their main findings were that overall metal levels were highest in the non-SCZ cigarette smoking group. SCZ patients that smoked were second highest, and third was non-smoking healthy controls. Importantly, cigarette brands themselves contain highly variable metal concentrations even within batches, which was not controlled for during the study [77].

Methodological limitations—including less sensitive detection techniques, incomplete reporting of analytical methods, and restricted study populations—may influence the observed associations between metal exposures and SCZ. Karim et al. [42] and Rahman et al., [40] utilized flame atomic absorption spectrometry (FAAS), a method that is less sensitive than GFAAS or ICP-MS for detecting trace metals in biological samples. The former did not observe an association between non-essential metal exposure and SCZ (SCZ), while the latter did observe elevated Cd. However, given the chosen detection methods higher detection limits and greater susceptibility to matrix interference, the use of FAAS may have limited the ability to detect subtle exposure differences between groups. Of note, Arinola et al., [9] did not specify what type of absorbance atomic absorption spectroscopy (AAS) they used (GFAAS or FAAS). Additionally, Rahman et al., [40] did not include women in their study, limiting its generalizability. Furthermore, Rahman et al., [40] chose to use scalp hair as their biological sampling specimen. Cd excretion through hair has been shown to have variable results, with Cd exposure having to have taken place > 1 year before it is detectable in hair [78]. Future studies should integrate ICP-MS–based blood metal assessments with scalp hair analyses, alongside detailed exposure histories and timing of diagnosis and sampling, to better evaluate the validity and temporal resolution of hair as a metal biomarker in SCZ populations.

Regarding the two studies by Opler et al., [29] and Opler et al., [30], there are potential, though unlikely confounding variables regarding the use of δ-ALA as a proxy for Pb levels. While δ-ALA is a validated proxy due to lead’s inhibition of the ALAD enzyme, it can also be elevated in individuals with porphyria, a rare hereditary condition. The authors did not indicate whether subjects were screened for porphyria. Direct measurement of blood lead via mass spectrometry would have been preferable, though this was not possible given the retrospective use of bio-banked serum samples from a 1959–1966 birth cohort. Like the cross-sectional studies, these prospective birth cohort studies [29, 30] and the retrospective cohort study by Modabbernia et al., [32] did not control for known environmental risk factors for SCZ. Although variables such as parental age, education, income, and substance use were included as covariates, key exposures like maternal infection, childhood adversity, and nutrient deficiency were overlooked. The failure to incorporate these relevant environmental variables limits the interpretability of the associations observed.

Several other important limitations were identified across the case control and cross-sectional case control studies included in this review. First, all studies relied on patient populations already diagnosed with SCZ and receiving care in medical or research facilities. This introduces selection bias, as such populations are likely skewed toward individuals with sufficient socioeconomic means or geographic proximity to care. As a result, these studies may underrepresent individuals from remote or lower-income backgrounds, where metal exposure—particularly through agricultural or industrial pathways—may be more prevalent. This bias could attenuate observed associations, pushing results toward the null.

Second, the cross-sectional design of several studies precludes determination of temporality; it remains unclear whether metal exposure preceded the onset of SCZ, thereby limiting causal inference. This limitation may be particularly relevant given evidence that individuals with SCZ may have impaired glutathione production, a critical endogenous metal-chelating and antioxidant [73, 79, 80]. Reduced glutathione capacity could promote disproportionate metal accumulation over time in individuals with SCZ due to impaired detoxification abilities. Consequently, measured metal concentrations at the time of bio-sampling may not accurately reflect exposure levels at or near disease onset, particularly if a substantial interval elapsed between diagnosis and sample collection.

Third, variability in diagnostic criteria and assessment of symptom severity across studies limits the generalizability of findings. SCZ is a highly heterogeneous disorder rather than a single uniform disease entity, with distinct symptom dimensions (e.g., positive, negative, and cognitive symptoms) differentially represented across individuals. It is plausible that specific metals may preferentially influence particular symptom domains [47], complicating interpretation when diagnostic frameworks differ. Notably, multiple diagnostic criteria were used across the 15 studies included in this review, introducing additional methodological heterogeneity.

Finally, none of the studies in this review accounted for other well-established environmental risk factors for SCZ, such as early-life trauma, prenatal infections, gestational micronutrient deficiencies, adolescent cannabis use, winter birth, or *Toxoplasma gondii* exposure [25–28]. Cannabis, for instance, can be a source of metal exposure [81]. The omission of these potential confounders diminishes the internal validity of the findings.

In sum, the methodological constraints across both cross-sectional and retrospective cohort studies reduce confidence in the causal interpretation of the relationship between metal exposure and SCZ. Future research should employ prospective designs, improve biomarker specificity, and rigorously account for the broader array of environmental risk factors known to contribute to SCZ pathogenesis. Doing so will help clarify the true extent to which metals, such as Pb, play a causal role in disease development.

### Biological Plausibility

The goal of this review was to examine the association between SCZ with non-essential, likely anthropogenically produced metals with no known biologically relevant use. However, in gathering this information, imbalances in essential metals such as Fe, Mn, Zn, Se, and Ca in SCZ were observed in numerous studies and therefore reported.

The relationship between SCZ, Zn, and Pb is complex and well observed [9, 82–85]. There exists strong biological plausibility that metal exposures could contribute to SCZ disease onset or exacerbation by interrupting NMDAr function, possibly through mimicry of Ca and Zn [86–88]. At resting membrane potential, NMDAr channels are blocked by magnesium ions; depolarization—primarily triggered by sodium influx through AMPArs—removes this block and enables Ca entry, a critical event for synaptic plasticity and downstream signaling. Magnesium dislodging is possible upon agonist binding of glutamate and glycine. Notably, the NR2A subunit possesses a cysteine-rich high-affinity Zn-binding domain, which is less influential in NR2B, potentially rendering NR2A-containing receptors more vulnerable to inhibition by thiophilic environmental toxicants such as lead [89]. Additionally, NR2A and NR2B subunits differ in their functional properties: NR2A-containing receptors exhibit higher activation probability and faster deactivation kinetics than NR2B-containing ones [90–92].

**Fig. 2:**
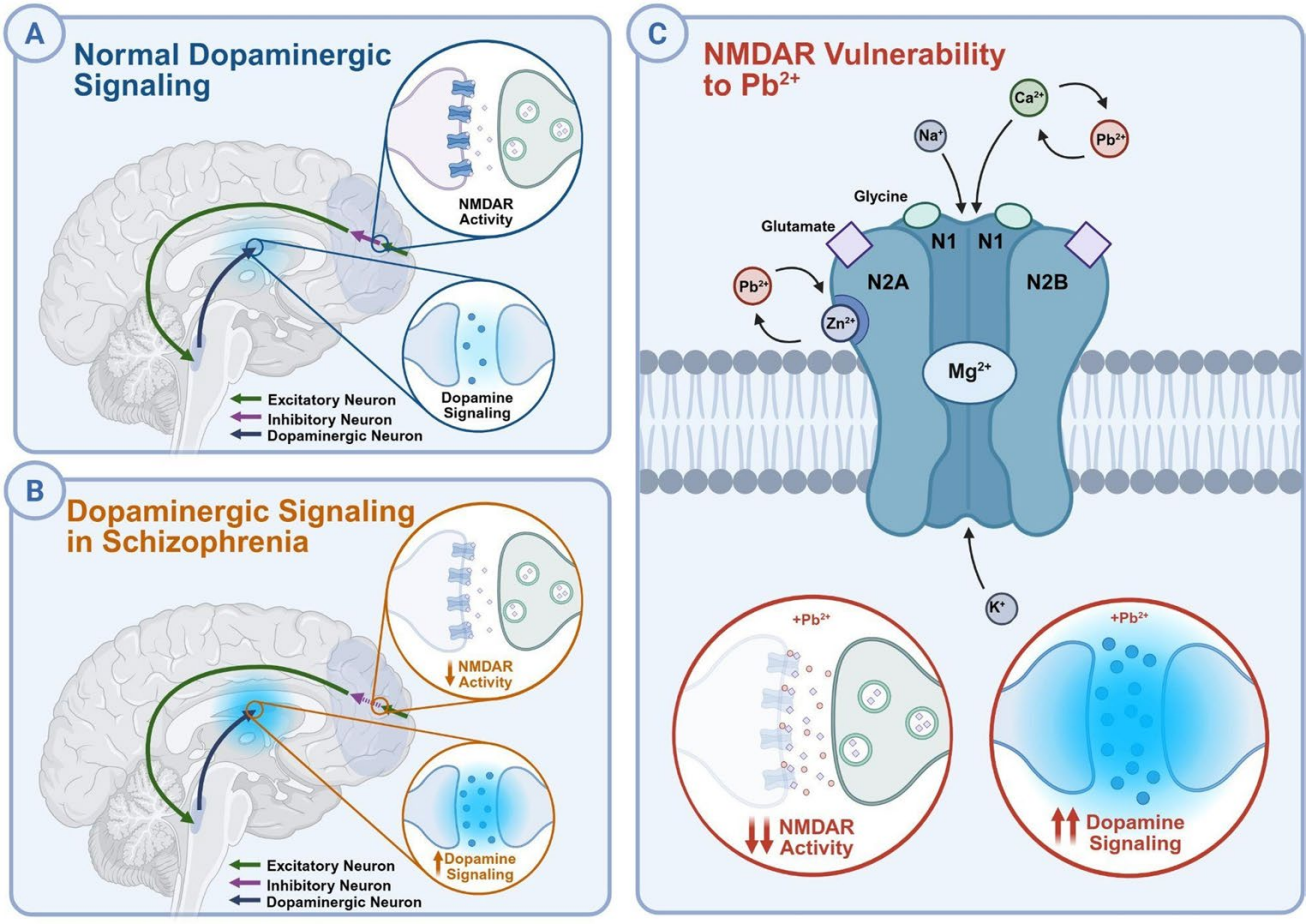
Schematic of NMDA receptor subunit composition and possible lead (Pb²⁺) interference. (**A**) Schematic of normal dopaminergic signaling in the brain. (**B**) In SCZ, NMDA receptor hypofunction in inhibitory neurons results in dopaminergic overexcitation. (**C**) NMDA receptor is vulnerable to Pb exposure due to its reliance on Zn and Ca, which have similarities to Pb. Lead exposure may exacerbate or trigger NMDA receptor hypofunction in SCZ patients, resulting in greater dopaminergic overexcitability

Zn plays an important role at the NMDAr, modulating its function by binding to a cysteine-rich binding pocket within the NR2A subunit, thereby inhibiting receptor activity in a concentration-dependent manner [93]. Agonist binding of the NMDAr, in conjunction with a dislodged magnesium ion from the NMDAr Ca channel due to sufficient membrane depolarization via influx of Na ions through AMPArs, induces a conformational change that displaces Zn from the allosteric Zn-binding site, enabling Ca influx, forming the basis of excitatory neurotransmission [23, 94] (Fig. 2C).

Thus, Pb may pose a unique threat to NMDAr function because of its tendency to mimic both Ca and Zn due to similar electron orbital configuration and atomic radii [24] (Fig. 2C). Moreover, Pb’s high affinity for thiol (–SH) groups on cysteine residues may increase the likelihood that it can compete for or disrupt occupancy of the receptor’s cysteine-rich regulatory pocket, thereby altering allosteric modulation. Collectively, these properties render the NMDAr—whose function is tightly regulated by Ca and Zn—particularly susceptible to Pb interference, potentially in a subunit composition–dependent manner; however, conflicting experimental findings have made this hypothesis controversial [86, 95–97]. NMDAr hypofunction has been implicated in core symptoms of SCZ, including hallucinations, paranoia, cognitive impairments, and psychosis [88, 98]. As previously discussed, mutations in *GRIN2A*—the gene encoding the NR2A subunit—are strongly implicated in SCZ. Many of these mutations reduce NR2A expression or impair its function. Given the unique susceptibility of the NR2A subunit to Pb inhibition, individuals with *GRIN2A* variants may be at particularly elevated risk for Pb-induced SCZ, as their already diminished NMDAr signaling may be pushed past a pathological threshold. Thus, the convergence of molecular vulnerability, genetic predisposition, and environmental exposure strengthens the case for a potentially causal relationship between lead exposure and SCZ.

In normal dopaminergic signaling, GABAergic neurons are stimulated by glutamate through proper NMDAr function, which in turn inhibit dopaminergic neurons projecting from the ventral tegmental area into the prefrontal cortex, inducing a default state of inhibition [98] (Fig. 2A). In SCZ, the glutamate hypothesis posits that dysfunction in glutamatergic signaling—specifically through hypofunction of NMDArs—contributes to the disorder’s pathophysiology by disrupting excitatory-inhibitory balance and impairing synaptic plasticity. NMDAr hypofunction in inhibitory neurons in the prefrontal cortex (PFC) may lead to hypoactive parvalbumin-positive GABAergic interneurons, resulting in *disinhibition* of dopaminergic neurons in the PFC, inducing overexcitation and excessive dopaminergic stimulation **(**Fig. 2B**)**. Evidence suggests that excessive dopaminergic activity in the PFC is responsible for producing the positive symptoms associated with the disease. Consequently, treatment for SCZs frequently involves the use of antipsychotics, most of which function by blocking dopamine receptors.

### Strengths and Limitations

To our knowledge, this is the first review to systematically examine the relationship between specific non-essential metal exposures and SCZ incidence, using predefined search terms and leveraging multiple databases, including PubMed and Google Scholar. Previous reviews have examined the association of metal exposures on multiple psychiatric phenotypes with vastly different biological mechanisms, making it difficult to hypothesize a mechanism of disease onset or exacerbation in response to exposure. The present review brings together consistent findings across independently conducted epidemiological studies and provides hypothesized biological mechanisms that could explain these findings.

The main limitations of this review include a generally small number of studies examined due to the specificity of the research question. Additionally, the studies included in this review did not account for other known environmental triggers for SCZ in their analyses, introducing a possible false attribution of disease phenotype to metal exposures when it may have been due to a separate, unaccounted for environmental trigger. Another limitation of both this review and the studies analyzed is the lack of consideration for potential sex-specific differences in susceptibility to metal-induced neurotoxicity. Additionally, variation in the operationalization of schizophrenia diagnostic criteria, symptom severity classification, and cutoffs for “high” versus “low” Pb exposure across studies hinders cross-study comparability and constrains the generalizability of findings. Also, the potential for publication bias must be acknowledged. This review identified only studies reporting at least one significant association, which may obscure the presence of unpublished or unreported negative findings due to publication bias.

Lastly, a major limitation in this field is the difficulty in choosing the biosample that most accurately reflects metal burden at the time of SCZ diagnosis. Future studies should account for the temporal gap between SCZ diagnosis and biosample collection, as detectable metal concentrations for certain metals (e.g., Pb) may fluctuate over time in different tissues (e.g., blood versus bone). Therefore, the choice of biosample (e.g., whole blood, hair, bone, or blood serum) should be carefully considered for each metal, given their distinct absorption, distribution, metabolism, and excretion (ADME) properties, which influence relative tissue accumulation and measured abundance.

## Conclusion

A review was conducted to investigate the association between metal exposure and SCZ. Of the 15 studies included in this review, 12 reported a positive association between non-essential metal exposure and SCZ [9, 29, 30, 32–36, 38–41]. Ten studies measured Pb in biological samples, eight of which showed a positive association between Pb and SCZ, representing the strongest positive association among the metals reviewed with SCZ followed by Cd, which was positively associated with SCZ in four of nine studies [9, 29, 30, 32, 34, 38, 43]. These findings could be consistent with biological mechanisms of how Pb interacts with the NMDAr, a receptor known for its role in contributing to SCZ phenotypes during hypofunction. Positive associations were found in other non-essential metals including Cr, As, U, W, Sb, Ba, and Cs. Also, in the process of this review, many essential metals were also noted to be dysregulated in individuals with SCZ; however, a formal systematic analysis on the epidemiological evidence of dysregulated essential metals and schizophrenia warrants its own separate investigation. Future research should analyze how the type and timing of biological sampling relative to diagnosis may affect metal concentrations. Additionally, future research scoring how SCZ symptom severity is affected by metal exposure ought to be explored. Finally, additional research integrating how genetic vulnerabilities to SCZ may affect metal absorption and detoxification ought to be conducted to better understand the public health thresholds for metal exposure in at risk populations.

## Key References

- Moghaddam B, Javitt D. From Revolution to Evolution: The Glutamate Hypothesis of Schizophrenia and its Implication for Treatment. *Neuropsychopharmacology* 2012; 37: 4–15.
  - ○ This review synthesizes the fields’ consensus on the glutamate hypothesis of schizophrenia.
- Guilarte TR, Miceli RC, Jett DA. Biochemical evidence of an interaction of lead at the zinc allosteric sites of the NMDA receptor complex: effects of neuronal development.*Neurotoxicology* 1995; 16: 63–71.
  - ○ This paper provides mechanistic evidence which suggests that lead (Pb) may disrupt NMDAr function by acting at the allosteric zinc binding pocket.
- Rabinowitz MB, Wetherill GW, Kopple JD. Kinetic analysis of lead metabolism in healthy humans. *J Clin Invest* 1976; 58: 260–270.
  - ○ This article provides foundational lead (Pb) metabolism kinetics.
- Hu H, Shih R, Rothenberg S, et al. The epidemiology of lead toxicity in adults: measuring dose and consideration of other methodologic issues. *Environ Health Perspect* 2007; 115: 455–462.
  - ○ This review discusses the importance of different epidemiological sampling methods in determining lead (Pb) body burden and potential challenges with common biomarkers used in research.

## Data Availability

No datasets were generated or analysed during the current study.

## References

[1] Briffa J, Sinagra E, Blundell R. Heavy metal pollution in the environment and their toxicological effects on humans. Heliyon. 2020;6:e04691. 32964150 10.1016/j.heliyon.2020.e04691 PMC7490536

[2] Koyama H, Kamogashira T, Yamasoba T. Heavy Metal Exposure: Molecular Pathways, Clinical Implications, and Protective Strategies. Antioxidants. 2024;13:76. 38247500 10.3390/antiox13010076 PMC10812460

[3] Tchounwou PB, Yedjou CG, Patlolla AK, et al. Heavy Metals Toxicity and the Environment. EXS. 2012;101:133–64. 22945569 10.1007/978-3-7643-8340-4_6 PMC4144270

[4] Alengebawy A, Abdelkhalek ST, Qureshi SR, et al. Heavy Metals and Pesticides Toxicity in Agricultural Soil and Plants: Ecological Risks and Human Health Implications. Toxics. 2021;9:42. 33668829 10.3390/toxics9030042 PMC7996329

[5] US EPA R 09. Abandoned Mines Cleanup, https://www.epa.gov/navajo-nation-uranium-cleanup/aum-cleanup (2016, accessed 26 April 2024).

[6] Levin R, Zilli Vieira CL, Rosenbaum MH, et al. The urban lead (Pb) burden in humans, animals and the natural environment. Environ Res. 2021;193:110377. 33129862 10.1016/j.envres.2020.110377 PMC8812512

[7] Mielke HW, Reagan PL. Soil is an important pathway of human lead exposure. Environ Health Perspect. 1998;106:217–29. 9539015 10.1289/ehp.98106s1217 PMC1533263

[8] Shim YK, Lewin MD, Ruiz P, et al. Prevalence and associated demographic characteristics of exposure to multiple metals and their species in human populations: the United States NHANES, 2007–2012. J Toxicol Environ Health A. 2007;80:502–12. 10.1080/15287394.2017.1330581 PMC5693367 28703686

[9] Arinola G, Idonije B, Akinlade K, et al. Essential trace metals and heavy metals in newly diagnosed schizophrenic patients and those on anti-psychotic medication. J Res Med Sci Off J Isfahan Univ Med Sci. 2010;15:245–9. PMC3082825 21526091

[10] Tyler CR, Allan AM. The effects of arsenic exposure on neurological and cognitive dysfunction in human and rodent studies: a review. Curr Environ Health Rep. 2014;1:132–47. 24860722 10.1007/s40572-014-0012-1 PMC4026128

[11] Saha S, Chant D, Welham J, et al. A Systematic Review of the Prevalence of Schizophrenia. PLOS Med. 2005;2:e141. 15916472 10.1371/journal.pmed.0020141 PMC1140952

[12] Charlson FJ, Ferrari AJ, Santomauro DF, et al. Global epidemiology and burden of schizophrenia: findings from the Global Burden of Disease Study 2016. Schizophr Bull. 2016;44:1195–203. 10.1093/schbul/sby058 PMC6192504 29762765

[13] Owen MJ, Sawa A, Mortensen PB. Schizophrenia. Lancet. 2016;388:86–97. 26777917 10.1016/S0140-6736(15)01121-6 PMC4940219

[14] Rahman T, Lauriello J. Schizophrenia: An Overview. Focus J Life Long Learn Psychiatry. 2016;14:300–7. 10.1176/appi.focus.20160006 PMC6526801 31975810

[15] Petronis A. The origin of schizophrenia: genetic thesis, epigenetic antithesis, and resolving synthesis. Biol Psychiatry. 2004;55:965–70. 15121478 10.1016/j.biopsych.2004.02.005

[16] Poltavskaya EG, Fedorenko OY, Kornetova EG, et al. Study of early onset schizophrenia: associations of GRIN2A and GRIN2B polymorphisms. Life. 2021;11:997. 34685369 10.3390/life11100997 PMC8540378

[17] Harrison PJ, Bannerman DM. GRIN2A (NR2A): a gene contributing to glutamatergic involvement in schizophrenia. Mol Psychiatry. 2023;28:3568–72. 37736757 10.1038/s41380-023-02265-y PMC10730418

[18] Millar JK, Wilson-Annan JC, Anderson S, et al. Disruption of two novel genes by a translocation co-segregating with schizophrenia. Hum Mol Genet. 2000;9:1415–23. 10814723 10.1093/hmg/9.9.1415

[19] Collingridge GL, Kehl SJ, McLennan H. Excitatory amino acids in synaptic transmission in the Schaffer collateral-commissural pathway of the rat hippocampus. J Physiol. 1983;334:33–46. 6306230 10.1113/jphysiol.1983.sp014478 PMC1197298

[20] Bliss TVP, Lømo T. Long-lasting potentiation of synaptic transmission in the dentate area of the anaesthetized rabbit following stimulation of the perforant path. J Physiol. 1973;232:331–56. 4727084 10.1113/jphysiol.1973.sp010273 PMC1350458

[21] Balu DT. The NMDA Receptor and Schizophrenia: From Pathophysiology to Treatment. Adv Pharmacol San Diego Calif. 2016;76:351–82. 10.1016/bs.apha.2016.01.006 PMC5518924 27288082

[22] Stys PK, You H, Zamponi GW. Copper-dependent regulation of NMDA receptors by cellular prion protein: implications for neurodegenerative disorders. J Physiol. 2012;590:1357–68. 22310309 10.1113/jphysiol.2011.225276 PMC3382327

[23] Jewett BE, Thapa B, Physiology. NMDA Receptor. In: *StatPearls*. Treasure Island (FL): StatPearls Publishing. http://www.ncbi.nlm.nih.gov/books/NBK519495/ (2025, accessed 12 February 2026). 30137779

[24] Bressler JP, Goldstein GW. Mechanisms of lead neurotoxicity. Biochem Pharmacol. 1991;41:479–84. 1671748 10.1016/0006-2952(91)90617-e

[25] Moreno JL, Kurita M, Holloway T, et al. Maternal Influenza Viral Infection Causes Schizophrenia-Like Alterations of 5-HT2A and mGlu2 Receptors in the Adult Offspring. J Neurosci. 2011;31:1863–72. 21289196 10.1523/JNEUROSCI.4230-10.2011 PMC3037097

[26] Maxwell AM, Rao RB. Perinatal iron deficiency as an early risk factor for schizophrenia. Nutr Neurosci. 2022;25:2218–27. 34165398 10.1080/1028415X.2021.1943996 PMC8702576

[27] HALL W. Cannabis use and the risk of developing a psychotic disorder. World Psychiatry. 2008;7:68–71. 18560513 10.1002/j.2051-5545.2008.tb00158.x PMC2424288

[28] Torrey EF, Yolken RH. *Toxoplasma gondii* and schizophrenia. Emerg Infect Dis. 2003;9:1375–80. 14725265 10.3201/eid0911.030143 PMC3035534

[29] Opler MGA, Brown AS, Graziano J, et al. Prenatal lead exposure, delta-aminolevulinic acid, and schizophrenia. Environ Health Perspect. 2004;112:548–52. 15064159 10.1289/ehp.6777 PMC1241919

[30] Opler MGA, Buka SL, Groeger J, et al. Prenatal exposure to lead, δ-aminolevulinic acid, and schizophrenia: further evidence. Environ Health Perspect. 2008;116:1586–90. 19057716 10.1289/ehp.10464 PMC2592283

[31] Liu T, Lu Q-B, Yan L, et al. Comparative Study on Serum Levels of 10 Trace Elements in Schizophrenia. PLoS ONE. 2015;10:e0133622. 26186003 10.1371/journal.pone.0133622 PMC4505857

[32] Modabbernia A, Velthorst E, Gennings C, et al. Early-life metal exposure and schizophrenia: A proof-of-concept study using novel tooth-matrix biomarkers. Eur Psychiatry J Assoc Eur Psychiatr. 2016;36:1–6. 10.1016/j.eurpsy.2016.03.006 PMC5300790 27311101

[33] Li Z, Liu Y, Li X, et al. Association of Elements with Schizophrenia and Intervention of Selenium Supplements. Biol Trace Elem Res. 2018;183:16–21. 28812245 10.1007/s12011-017-1105-0

[34] Ma J, Yan L, Guo T, et al. Association of typical toxic heavy metals with schizophrenia. Int J Environ Res Public Health. 2019;16:4200. 31671526 10.3390/ijerph16214200 PMC6862006

[35] Ma J, Wang B, Gao X, et al. A comparative study of the typical toxic metals in serum by patients of schizophrenia and healthy controls in China. Psychiatry Res. 2018;269:558–64. 30199697 10.1016/j.psychres.2018.08.114

[36] Li J, Chen J, Shen B, et al. Association of non-essential metals with Chinese schizophrenia: a case-control study. Early Interv Psychiatry. 2024;18:615–23. 38339807 10.1111/eip.13505

[37] Ghaderi A, Khoshakhlagh AH, Gruszecka-Kosowska A, et al. Heavy metal concentrations and clinical symptoms in patients diagnosed with schizophrenia related to cigarette smoking. Sci Rep. 2024;14:15074. 38956098 10.1038/s41598-024-64333-9 PMC11219874

[38] Shen B, Lu R, Lv M, et al. Association between the levels of toxic heavy metals and schizophrenia in the population of Guangxi, China: A case-control study. Environ Pollut Barking Essex 1987. 2024;363:125179. 10.1016/j.envpol.2024.125179 39490508

[39] Ray A, Tomo S, Yadav D, et al. Exploring the association between heavy metals, TNF-α regulation, and cognitive dysfunction in schizophrenia. Biol Trace Elem Res. 2026;204:639–49. 40533715 10.1007/s12011-025-04701-2

[40] Rahman MA, Azad MAK, Hossain MI, et al. Zinc, Manganese, Calcium, Copper, and Cadmium Level in Scalp Hair Samples of Schizophrenic Patients. Biol Trace Elem Res. 2009;127:102–8. 18810332 10.1007/s12011-008-8230-8

[41] Almulla AF, Moustafa SR, Al-Dujaili AH, et al. Lowered serum cesium levels in schizophrenia: association with immune-inflammatory biomarkers and cognitive impairments. Rev Bras Psiquiatr Sao Paulo Braz 1999. 2021;43:131–7. 10.1590/1516-4446-2020-0908 PMC8023164 32556004

[42] Karim P, Hossain MI, Sadat AN, et al. Serum levels of Cadmium, Calcium, Lead and Iron in Schizophrenic Patients. Dhaka Univ J Pharm Sci. 2006;5:9–13.

[43] Nawaz R, Zahir E, Siddiqui S, et al. The role of trace metals and environmental factors in the onset and progression of schizophrenia in Pakistani population. World J Neurosci. 2014;04:450–60.

[44] Li X, Sundquist J, Hemminki K, et al. Familial Risks of Psychotic Disorders and Schizophrenia among Siblings Based on Hospitalizations in Sweden. Psychiatry Res. 2009;166:1–6. 19208442 10.1016/j.psychres.2007.12.003 PMC2696603

[45] Stanley PC, Wakwe VC. Toxic trace metals in the mentally ill patients. Niger Postgrad Med J. 2002;9:199–204. 12690679

[46] Sallmén M, Suvisaari J, Lindbohm M-L, et al. Paternal occupational lead exposure and offspring risks for schizophrenia. Schizophr Res. 2016;176:560–5. 27318522 10.1016/j.schres.2016.06.004

[47] Todorić Laidlaw I, Mimica N, Momčilović B, et al. Trace elements concentrations association with schizophrenia symptoms; A cross-sectional study in Croatia. Psychiatr Danub. 2018;30:164–71. 29930226 10.24869/psyd.2018.164

[48] Romanò A, Pagiatakis C, Gornati R, et al. Epigenetics: a link between toxicants and diseases. iScience. 2025;28:112613. 40487436 10.1016/j.isci.2025.112613 PMC12143666

[49] Adesina KE, Burgos CJ, Grier TR, et al. Ways to Measure Metals: From ICP-MS to XRF. Curr Environ Health Rep. 2025;12:7. 39865194 10.1007/s40572-025-00473-y PMC11913532

[50] Trzcinka-Ochocka M, Brodzka R, Janasik B. Useful and Fast Method for Blood Lead and Cadmium Determination Using ICP‐MS and GF‐AAS; Validation Parameters. J Clin Lab Anal. 2014;30:130–9. 25425387 10.1002/jcla.21826 PMC6807167

[51] Sperling M. Flame and Graphite Furnace Atomic Absorption Spectrometry in Environmental Analysis. In: Meyers RA, editor. Encyclopedia of Analytical Chemistry. Wiley; 2006. pp. 1–69. 10.1002/9780470027318.a0805

[52] Bowler RM, Mergler D, Sassine MP, et al. Neuropsychiatric effects of manganese on mood. Neurotoxicology. 1999;20:367–78. 10385897

[53] Verhoeven WM, Egger JI, Kuijpers HJ. Manganese and acute paranoid psychosis: a case report. J Med Case Rep. 2011;5:146. 21486469 10.1186/1752-1947-5-146 PMC3090741

[54] Ma J, Yan L, Guo T, et al. Association between serum essential metal elements and the risk of schizophrenia in China. Sci Rep. 2020;10:10875. 10.1038/s41598-020-66496-7 PMC7335092 32620780

[55] Bouchard M, Mergler D, Baldwin M, et al. Neuropsychiatric symptoms and past manganese exposure in a ferro-alloy plant. Neurotoxicology. 2007;28:290–7. 16962176 10.1016/j.neuro.2006.08.002

[56] Goyer RA. Nutrition and metal toxicity. Am J Clin Nutr. 1995;61:S646–50. 10.1093/ajcn/61.3.646S 7879732

[57] Tsaih SW, Korrick S, Schwartz J, et al. Influence of bone resorption on the mobilization of lead from bone among middle-aged and elderly men: the Normative Aging Study. Environ Health Perspect. 2001;109:995–9. 11675263 10.1289/ehp.01109995 PMC1242074

[58] Silbergeld EK, Schwartz J, Mahaffey K. Lead and osteoporosis: mobilization of lead from bone in postmenopausal women. Environ Res. 1988;47:79–94. 3168967 10.1016/s0013-9351(88)80023-9

[59] Populations NRC (US) C on ML in C, editor. Biologic Markers of Lead Toxicity. In: *Measuring Lead Exposure in Infants, Children, and Other Sensitive Populations*. National Academies Press (US). https://www.ncbi.nlm.nih.gov/books/NBK236462/ (1993, accessed 6 February 2026). 25144057

[60] Rabinowitz MB, Wetherill GW, Kopple JD. Kinetic analysis of lead metabolism in healthy humans. J Clin Invest. 1976;58:260–70. 783195 10.1172/JCI108467 PMC333178

[61] Ziegler EE, Edwards BB, Jensen RL, et al. Absorption and Retention of Lead by Infants. Pediatr Res. 1978;12:29–34. 643372 10.1203/00006450-197801000-00008

[62] Alexander FW. The Uptake of Lead by Children in Differing Environments. Environ Health Perspect. 1974;7:155–9. 4831138 10.1289/ehp.747155 PMC1475120

[63] Bergdahl IA, Grubb A, Schütz A, et al. Lead binding to delta-aminolevulinic acid dehydratase (ALAD) in human erythrocytes. Pharmacol Toxicol. 1997;81:153–8. 9353844 10.1111/j.1600-0773.1997.tb02061.x

[64] Lead poisoning. https://www.who.int/news-room/fact-sheets/detail/lead-poisoning-and-health (accessed 19 August 2024).

[65] Lead. July. *National Institute of Environmental Health Sciences*, https://www.niehs.nih.gov/health/topics/agents/lead (accessed 30 2024).

[66] Chettle DR, Scott MC, Somervaille LJ. Lead in bone: sampling and quantitation using K X-rays excited by 109Cd. Environ Health Perspect. 1991;91:49–55. 2040251 10.1289/ehp.919149 PMC1519364

[67] Gulson BL, Mizon KJ, Korsch MJ, et al. Mobilization of lead from human bone tissue during pregnancy and lactation–a summary of long-term research. Sci Total Environ. 2003;303:79–104. 12568766 10.1016/s0048-9697(02)00355-8

[68] Goldman RH, White R, Kales SN, et al. Lead poisoning from mobilization of bone stores during thyrotoxicosis. Am J Ind Med. 1994;25:417–24. 8160659 10.1002/ajim.4700250309

[69] Cake KM, Bowins RJ, Vaillancourt C, et al. Partition of circulating lead between serum and red cells is different for internal and external sources of lead. Am J Ind Med. 1996;29:440–5. 8732917 10.1002/(SICI)1097-0274(199605)29:5<440::AID-AJIM2>3.0.CO;2-Q

[70] Hu H, Shih R, Rothenberg S, et al. The epidemiology of lead toxicity in adults: measuring dose and consideration of other methodologic issues. Environ Health Perspect. 2007;115:455–62. 17431499 10.1289/ehp.9783 PMC1849918

[71] Smith D, Hernandez-Avila M, Téllez-Rojo MM, et al. The relationship between lead in plasma and whole blood in women. Environ Health Perspect. 2002;110:263–8. 11882477 10.1289/ehp.02110263 PMC1240766

[72] Lukács M, Csilla Pálinkás D, Szunyog G, et al. Metal binding ability of small peptides containing cysteine residues. ChemOpen. 2021;10:451–63. 10.1002/open.202000304 PMC8028610 33830669

[73] Jozefczak M, Remans T, Vangronsveld J, et al. Glutathione Is a Key Player in Metal-Induced Oxidative Stress Defenses. Int J Mol Sci. 2012;13:3145–75. 22489146 10.3390/ijms13033145 PMC3317707

[74] Comer AL, Carrier M, Tremblay M-È, et al. The inflamed brain in schizophrenia: the convergence of genetic and environmental risk factors that lead to uncontrolled neuroinflammation. Front Cell Neurosci. 2020. 10.3389/fncel.2020.00274. 33061891 10.3389/fncel.2020.00274 PMC7518314

[75] Doorduin J, de Vries EFJ, Willemsen ATM, et al. Neuroinflammation in Schizophrenia-Related Psychosis: A PET Study. J Nucl Med. 2009;50:1801–7. 19837763 10.2967/jnumed.109.066647

[76] Chibowska K, Baranowska-Bosiacka I, Falkowska A, et al. Effect of lead (Pb) on inflammatory processes in the brain. Int J Mol Sci. 2016;17:2140. 27999370 10.3390/ijms17122140 PMC5187940

[77] Sandal S, Verghese PS, Taneja A, et al. Cigarettes as a source of heavy metal toxicity: evaluating human health risks. Discov Public Health. 2025;22:311.

[78] Ellis KJ, Yasumura S, Cohn SH. Hair cadmium content: is it a biological indicator of the body burden of cadmium for the occupationally exposed worker? Am J Ind Med. 1981;2:323–30. 7048908 10.1002/ajim.4700020404

[79] Chowdari KV, Bamne MN, Nimgaonkar VL. Genetic Association Studies of Antioxidant Pathway Genes and Schizophrenia. Antioxid Redox Signal. 2011;15:2037–45. 20673164 10.1089/ars.2010.3508 PMC3159115

[80] Belcastro M, Marino T, Russo N, et al. The role of glutathione in cadmium ion detoxification: Coordination modes and binding properties – A density functional study. J Inorg Biochem. 2009;103:50–7. 18951636 10.1016/j.jinorgbio.2008.09.002

[81] McGraw KE, Nigra AE, Klett J, et al. Blood and urinary metal levels among exclusive marijuana users in NHANES (2005–2018). Environ Health Perspect. 2005;131:87019. 10.1289/EHP12074 PMC10467359 37646523

[82] Guilarte TR. Pb2 + inhibits NMDA receptor function at high and low affinity sites: developmental and regional brain expression. Neurotoxicology. 1997;18:43–51. 9215987

[83] Guilarte TR, Miceli RC, Jett DA. Biochemical evidence of an interaction of lead at the zinc allosteric sites of the NMDA receptor complex: effects of neuronal development. Neurotoxicology. 1995;16:63–71. 7603646

[84] Petrilli MA, Kranz TM, Kleinhaus K, et al. The Emerging Role for Zinc in Depression and Psychosis. Front Pharmacol. 2017;8:414. 28713269 10.3389/fphar.2017.00414 PMC5492454

[85] Ordemann JM, Austin RN. Lead neurotoxicity: exploring the potential impact of lead substitution in zinc-finger proteins on mental health. 10.1039/c5mt00300h (accessed 6 February 2026). 10.1039/c5mt00300h 26745006

[86] Neal AP, Worley PF, Guilarte TR. Lead exposure during synaptogenesis alters NMDA receptor targeting via NMDA receptor inhibition. Neurotoxicology. 2011;32:281–9. 21192972 10.1016/j.neuro.2010.12.013 PMC3049857

[87] Vallée A. Neuroinflammation in schizophrenia: the key role of the WNT/β-catenin pathway. Int J Mol Sci. 2022;23:2810. 35269952 10.3390/ijms23052810 PMC8910888

[88] Olney JW, Farber NB. Glutamate receptor dysfunction and schizophrenia. Arch Gen Psychiatry. 1995;52:998–1007. 7492260 10.1001/archpsyc.1995.03950240016004

[89] Neal AP, Guilarte TR. Molecular neurobiology of lead (Pb2+): effects on synaptic function. Mol Neurobiol. 2010;42:151–60. 21042954 10.1007/s12035-010-8146-0 PMC3076195

[90] Chen N, Luo T, Raymond LA. Subtype-dependence of NMDA receptor channel open probability. J Neurosci Off J Soc Neurosci. 1999;19:6844–54. 10.1523/JNEUROSCI.19-16-06844.1999 PMC6782868 10436042

[91] Erreger K, Geballe MT, Kristensen A, et al. Subunit-specific agonist activity at NR2A-, NR2B-, NR2C-, and NR2D-containing N-methyl-D-aspartate glutamate receptors. Mol Pharmacol. 2007;72:907–20. 17622578 10.1124/mol.107.037333

[92] Vicini S, Wang JF, Li JH, et al. Functional and pharmacological differences between recombinant N-methyl-D-aspartate receptors. J Neurophysiol. 1998;79:555–66. 9463421 10.1152/jn.1998.79.2.555

[93] Paoletti P, Perin-Dureau F, Fayyazuddin A, et al. Molecular organization of a zinc binding N-terminal modulatory domain in a NMDA receptor subunit. Neuron. 2000;28:911–25. 11163276 10.1016/s0896-6273(00)00163-x

[94] Kandel ER, Dudai Y, Mayford MR. The molecular and systems biology of memory. Cell. 2014;157:163–86. 24679534 10.1016/j.cell.2014.03.001

[95] Gavazzo P, Zanardi I, Baranowska-Bosiacka I, et al. Molecular determinants of Pb^2+^ interaction with NMDA receptor channels. Neurochem Int. 2008;52:329–37. 17706324 10.1016/j.neuint.2007.07.003

[96] Lasley SM, Gilbert ME. Lead inhibits the rat N-methyl-d-aspartate receptor channel by binding to a site distinct from the zinc allosteric site. Toxicol Appl Pharmacol. 1999;159:224–33. 10486309 10.1006/taap.1999.8743

[97] Alkondon M, Costa ACS, Radhakrishnan V, et al. Selective blockade of NMDA-activated channel currents may be implicated in learning deficits caused by lead. FEBS Lett. 1990;261:124–30. 1689669 10.1016/0014-5793(90)80652-y

[98] Moghaddam B, Javitt D. From Revolution to Evolution: The Glutamate Hypothesis of Schizophrenia and its Implication for Treatment. Neuropsychopharmacology. 2012;37:4–15. 21956446 10.1038/npp.2011.181 PMC3238069

